# Food preservation by *Larrea divaricata* extract: participation of polyphenols

**DOI:** 10.1002/fsn3.640

**Published:** 2018-05-16

**Authors:** Ignacio Peralta, Carla Marrassini, Rosana Filip, Maria R. Alonso, Claudia Anesini

**Affiliations:** ^1^ Universidad de Buenos Aires Consejo Nacional de Investigaciones Científicas y Técnicas (CONICET) Instituto de Química y Metabolismo del Fármaco (IQUIMEFA) Facultad de Farmacia y Bioquímica Universidad de Buenos Aires Buenos Aires Argentina

**Keywords:** antiglicant, antioxidant, food preservative, *Larrea divaricata* Cav.

## Abstract

The aim of this study was to evaluate the antioxidant and protease inhibitor capacities on eggs and milk protein of a nor‐dihydroguaiaretic (NDGA)‐standardized aqueous extract of *Larrea divaricata* (AE) and to analyze the participation of polyphenols as NDGA in these actions. NDGA was determined by high‐performance liquid chromatography; flavonoids and polyphenols were quantified by spectrophotometric methods as well as inhibition of lipid peroxidation, proteinase inhibitor capacity, advanced glycation end products (AGES) formation, and inhibition of albumin denaturation. The extract protected food for oxidative damage by preventing malondialdehyde formation in egg yolk and by preventing AGE formation in completely cooked eggs, also impeded albumin denaturation, and casein hydrolysis induced by trypsin and heat. Polyphenols, especially flavonoids and NDGA, were involved in these actions.

## 1 INTRODUCTION

Food is commonly submitted to oxidative processes which modify the appearance, flavor, aroma and also induce the formation of chemical compounds harmful for both animals and humans. The destruction of proteins, vitamins, and fatty acids decreases food values.

Synthetic antioxidants, used to prevent oxidative food damage, present many adverse effects. So nowadays, instead of using synthetic antioxidant, food manufacturers prefer natural and innocuousness products. Because of that, the antioxidant properties of plants are being studied exhaustively to be used as food preserver, in preference those that present polyphenols compounds.

The antioxidant activity is known to be related to the scavenging of reactive oxygen species (ROS). ROS participate in the oxidation of glucose and phospholipids giving glyco‐ and lipoxidated compounds such as malondialdehyde (MDA) and glucose oxidized derivatives. Finally, the interaction of these compounds with proteins originates advanced lipoxidation end products (ALEs) and advanced glycation end products (AGEs), respectively. It is known that ALE and AGE are involved not only in atherosclerosis, diabetes, arthritis, cancer, Alzheimer's disease, aging, neurodegenerative diseases (Yun‐Zhong, Sheng, & Guoyao, 2002) but also in food oxidation and attempt by this way to food preservation, inducing the formation of chemical compounds which may be harmful for both animals and humans. Lately, numerous studies have evidenced that diet is a significant exogenous source of highly reactive AGE and ALE. During food processing, food storage and cooking (by frying, roasting, and baking, including microwave usage) oxidation processes occur during which glyco‐ and lipoxidation compounds are formed. Then, these compounds interact with amino groups originating AGE through the Maillard (browning) reaction. Modern diets are largely heat‐processed, and as a result, they contain high levels of AGE. Especially, products of animal origin, which are rich in lipids and proteins, have a high AGE concentration and have the potential of forming new AGE throughout thermal processing (Goldberg et al., 2004; O'Brien & Morrissey, 1989). Other process that alters foods is protein degradation by enzymes called proteases as it occurs with casein and albumin from milk; proteins are also susceptible to hydrolysis by heat.

Both casein and albumin are very important proteins for human body. Casein is present in milk and milk‐derived product such as cheese, ice‐cream, yogurt, and it is used as dietary supplement to increase muscle formation in sporty man. By other way, albumin is important in the diet due to its high biological value as a source of essential amino acids. A source of albumin is eggs, milk, and meat.

*Larrea divaricata* Cav is a South American plant which is widely distributed in Argentina and used in folk medicine to treat inflammatory diseases. The aqueous extract (AE) of its leaves presents nor‐dihydroguaiaretic acid (NDGA) as the major polyphenolic compound. This extract is known to have well‐documented biological activities such as antitumor, immunomodulatory (Anesini, Ferraro, Lopéz, & Borda, 2001; Anesini et al., 1996; Martino, Súlsen, Alonso, & Anesini, 2013; Martino et al., 2016), antimicrobial (Anesini & Pérez, 1993; Stege et al., 2006), anti‐inflammatory (Davicino, Peralta, Martino, Alonso, & Anesini, 2015), and antioxidant. The antioxidant activity is related to the scavenging effect on radical DPPH and on the secretion of peroxidase in rat salivary glands (Alonso, Peralta, Lemos, Davicino, & Anesini, 2012; Anesini, Turner, Borda, Ferraro, & Coussio, 2004). It has also been demonstrated that the extract can prevent the oxidation of vitamin C in orange juice (Micucci, Alonso, Turner, Davicino, & Anesini,^2011^). To date, the preventive effect on food oxidative damage (inhibition of lipiperoxidation and AGEs formation in eggs during cooking) as well as the capacity of inhibition of protein hydrolysis by heat and by protease has not been determined with *L. divaricata* extracts.

Taking these data into account, the aim of this study was to determine the capacity of the extract to preserve food from oxidation and from heat and proteases damage. To do this, the effect of AE on peroxidation and AGE formation in fried eggs and on albumin heat‐induced denaturation and casein hydrolysis by trypsin was studied. Also, the polyphenols and flavonoids content were determined and the participation of NDGA in these actions was studied.

These results established the potential use of the extract as a new food preserver with antioxidant, anti‐AGE, and antiprotein hydrolysis activity to be added in foods for both human and animal consumption.

## 2 MATERIALS AND METHODS

### 2.1 Plant material and extract

Leaves of *L. divaricata* Cav. were collected in the province of Córdoba, Argentina, and identified using morphological, anatomical, and histochemical analysis. A voucher specimen (BAFC no. 38) was deposited in the Museum of Pharmacobotany, School of Pharmacy and Biochemistry, University of Buenos Aires.

To prepare the AE, the air‐dried leaves were extracted for 10 min with boiling distilled water (7.5%). The extract was then macerated, filtered, and lyophilized. The final yield was 26.6 g % of plant material. The AE was aliquoted and stored at −20°C until used (Anesini et al., 1996).

### 2.2 HPLC analysis

The high‐performance liquid chromatography analysis was performed in a Varian Pro Star instrument equipped with a Rheodyne injection valve (20 μl) and photodiode array detector set at 280 nm. A reversed‐phase column Phenomenex—C18 (2) Luna (250 mm × 4.6 mm and 5 μm pd) was used. As mobile phases, water and acetic acid (98:2, mobile phase A), and methanol and acetic acid (98:2, mobile phase B) were employed, the gradient conditions were 15% B to 40% B in 30 min; 40% B to 75% B in 10 min; 75% B to 85% B in 5 min, and 100% B in 5 min. The mobile phase B was kept at 100% for 10 min before restoring the initial conditions. Mobile phases were delivered with a flow rate of 1.2 ml/min. The chromatographic procedure was performed at room temperature (18–25°C). A pure NDGA standard (Sigma, USA) was used for identification and quantification by comparing retention times and by plotting peak areas, respectively (Davicino, Alonso, & Anesini, 2011).

### 2.3 Polyphenols and flavonoids determination

The total polyphenols content was determined by spectrophotometry according to the Folin–Ciocalteu's method using gallic acid as standard (Hosseinzadeh, Khorsandi, & Hemmaty, 2013). The lyophilized extract was weighted and dissolved in distilled water. Briefly, a sample of 1.0 ml of the extract was transferred to separate tubes containing 7.0 ml distilled water, 0.5 ml of Folin–Ciocalteu′s reagent, and 1.5 ml of a 20% sodium carbonate anhydrous solution. Solutions were then allowed to stand at room temperature for 60 min, and then, the absorbance at 765 nm was measured by employing a ultraviolet‐vis spectrophotometer. The concentration of polyphenols in samples was derived from a standard curve of gallic acid ranging from 10 to 50 μg/ml (Pearson's correlation coefficient: *r* ^2 ^= .9996). Results were expressed as mg GAE/g extract.

Total flavonoids were also determined on the AE by spectrophotometry. Briefly, the extract was mixed with aluminum trichloride and potassium acetate and incubated during 30 min. The absorbance at 415 nm was measured. Results were expressed as mg quercitrin/g extract derived from a calibration curve made with known concentrations of the flavonoid quercitrin (Sigma) (Dantas Fernandes et al., 2012).

### 2.4 Inhibition of lipid peroxidation in egg yolk

The antioxidant activity was determined in a model of phospholipids peroxidation in egg yolk (Kuppusamy, Indran, & Balraj, 2002). The extract or the NDGA in different concentrations was mixed with 1 ml of egg yolk, emulsified with 0.1 mol/L phosphate buffer (pH 7.4) to obtain a final concentration of 25 g/l, and 100 μl of 1000 μmol/L Fe^2+^. The mixture was incubated at 37°C for 1 hr and then treated with 0.5 ml of freshly prepared 15% trichloroacetic acid and 1.0 ml of 1% thiobarbituric acid. Reaction tubes were kept in a boiling water bath for 10 min. After cooling, tubes were centrifuged at 3500 g for 10 min to remove the precipitated protein. The formation of thiobarbituric acid reactive substances was measured at 532 nm. The control of oxidation consisted of buffered egg with Fe^2+^ only. The blank extract was prepared without egg yolk. Blank values were subtracted from each test tube. Butyl‐hydroxytoluene (BHT) (1000 μg/ml) was used as reference antioxidant drug. The percentage of peroxidation inhibition was calculated with the following equation: % inhibition: [(A0 – As)/A0] × 100, where A0 was the control of oxidation absorbance and As was the sample absorbance.

### 2.5 Inhibition of AGE formation in eggs during the cooking process

To determine AGE, eggs were subjected to a standard cooking method such as frying. Briefly, 2.5 ml of homogenized eggs plus 2.5 ml of water (positive oxidative control) and 2.5 ml of homogenized eggs plus 2.5 ml of AE (0.1, 1.0, 10 mg/ml) or 2.5 ml of NDGA (3 μg/ml) was fried with 1 ml of oil during 5 min at 70°C. Samples, obtained by this process, were then maintained at −20°C until used. To determine total AGE, 1 g of each sample was homogenized in 2 ml of PBS, centrifuged during 5 min at 900 g and the supernatant was used for the quantification of AGE. AGEs were determined by an indirect competitive enzyme immune assay kit (LAMIDER, Laboratorio Mexicano de Inmunoensayo y Diagnóstico de Referencia S.A. de C.V.). Total AGEs were calculated by interpolation in a calibration curve performed with AGE provided by the manufacturer.

### 2.6 Proteinase inhibitory action

The test was performed according to the method of Oyedepo (Oyedepo & Femurewa, 1995) with minor modifications. The reaction mixture (1 ml) contained 0.03 ml trypsin (0.3 μg), 0.5 ml 20 mmol/L Tris HCl buffer(pH 7.4), and 0.5 ml of the extract in different concentrations (10, 25, 50, 100 μg/ml). The mixture was incubated at 37°C for 5 min, and then 0.5 ml of 0.8% (w/v) casein was added. The mixture was incubated for an additional 20 min, and 1 ml of 70% perchloric acid was added to terminate the reaction. Cloudy suspension was centrifuged, and the absorbance of the supernatant was read at 280 nm against buffer as blank. The experiment was performed in quadruplicate. The inhibition of proteinase activity, as percentage, was calculated.

### 2.7 Inhibition of albumin denaturation

The method was performed according to Kumari, Yasmin, Hussain, & Babuselvam (2015) with minor modifications. The reaction mixture (0.5 ml) consisted of 0.40 ml bovine serum albumin (1% aqueous solution), 0.05 ml of the extract at different concentrations (1,000, 800, 600, and 400 μg/ml), and 0.05 ml of salicylic acid (inductor of protein denaturation). For control test, 0.05 ml distilled water was used instead of extracts while negative control test lacked salicylic acid. The samples were incubated at 37°C for 20 min and then heated at 57°C for 20 min. After cooling the samples, 2.5 ml phosphate buffer saline (pH 6.3) was added to each tube. Turbidity was measured spectrophotometrically at 660 nm. The inhibition of protein denaturation, as percentage, was calculated as follows.

$$\text{Percent inhibition}=\frac{\text{Abs Control ‐ Abs treated}}{\text{Abs Control}}\times 100.$$

The control represents 100% protein denaturation.

### 2.8 Statistical analysis

Data were expressed as the average of duplicate or triplicate values of three independent experiments. To compare two values, the Student's *t‐*test was used. Multiple comparisons were analyzed using ANOVA+ Newman–Keuls’ test or Tukey's test. A *p* ≤ .05 was considered statistically significant.

## 3 RESULTS AND DISCUSSION

**Figure 1:**
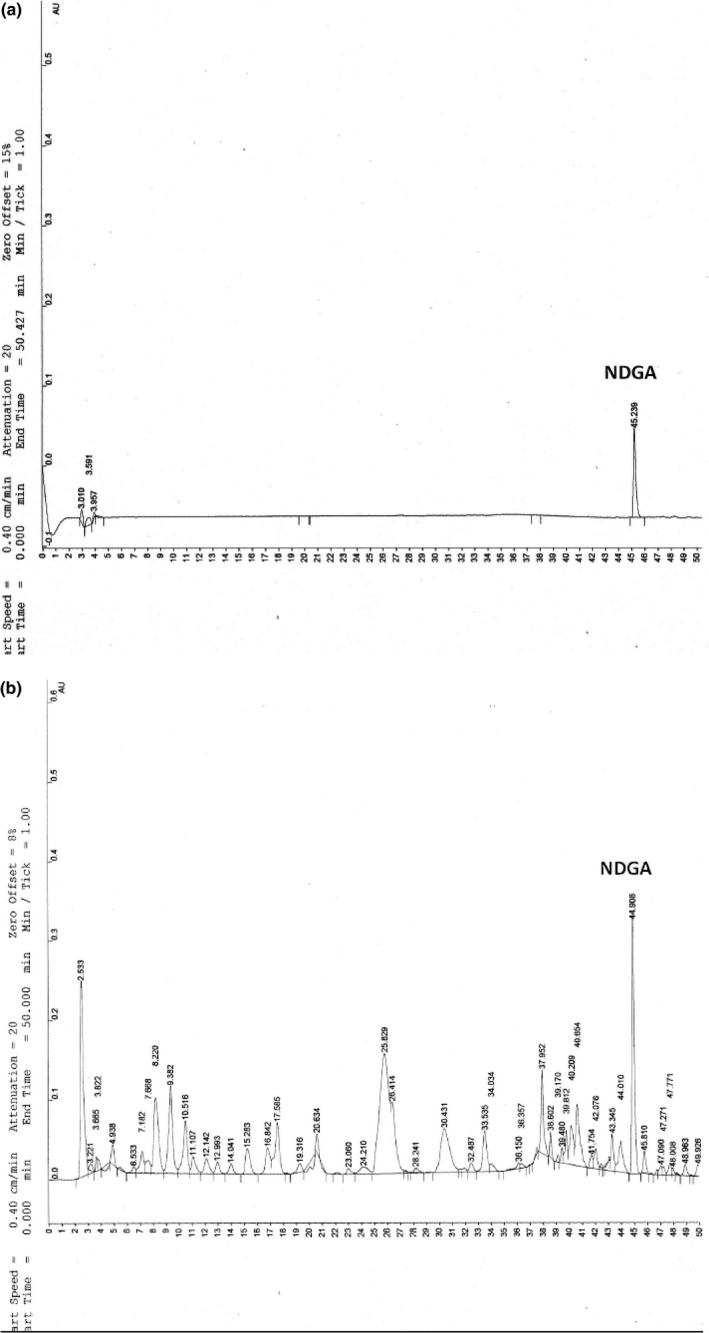
High‐performance liquid chromatography analysis of AE. (a) Chromatographic profile of NDGA standard. (b) Chromatogram corresponding to the AE; where the NDGA content represents the 0.3% w/w of the dry extract. For samples and standards, the same running conditions were used

First, NDGA was identified and quantified in the AE to work with a standardized extract and to study its participation in the protection of food. The chromatographic profile of AE is shown in Figure 1, where a peak corresponding to NDGA (0.30 g % w/w) with a retention time at 43.2 min can be observed. Also, total polyphenols and flavonoids were quantified in the extract (Table 1).

**Table 1:** Total polyphenols and flavonoids in the aqueous extract of *Larrea divaricata*

	Total polyphenols GAE (mg/g)	Total flavonoids Quercitrin (mg/g)
Aqueous extract	106.80 ± 4.30	24.63 ± 1.63

Results are expressed as mg GAE or quercitrin/g extract and represent mean ± SEM of two independent experiments performed in triplicate.

The presence of NDGA in the AE has previously been described (Anesini et al., 2001; Micucci et al., 2011). Despite its high solubility in ethanol, NDGA is poorly soluble in water. This differential solubility accounts for the low yield of this compound in the AE.

**Figure 2:**
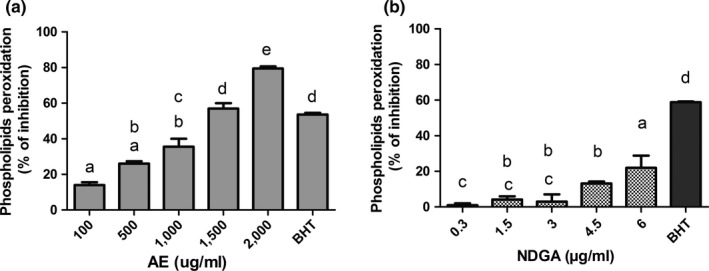
Effect of AE on phospholipids peroxidation in eggs. The inhibition of phospholipids peroxidation was determined as a measure of antioxidant activity. (a) Effect of AE (b) effect of NDGA. BHT: butyl‐hydroxytoluene was employed as the reference antioxidant. Results were expressed as mean ± SEM of three experiments made in triplicate. a, b, c, and d significant differences between treatments (ANOVA+ Newman–Keuls’ test)

Afterward, it was analyzed the extract's capacity of food preservation to oxidation, heat, and protease damage. As it can be seen in Figure 2a, the extract presented antioxidant activity, preventing the oxidation of phospholipids of egg yolk in a concentration–response‐dependent manner, this could be observed as a decrease in MDA formation, a marker of lipid peroxidation. The higher concentrations of the extract presented equal or more activity than BHT, the reference antioxidant. The NDGA was studied at the same concentration as that present in the extract. NDGA also inhibited oxidation in a concentration‐response relationship but exerted lower activity than the extract (Figure 2b). Nevertheless, the antioxidant activity of the extract could be partially related to the presence of NDGA as, at the same concentration present in the extract, it exerted low activity. In this way, it can be assumed that other polyphenols besides NDGA were involved in the antilipid peroxidative activity of the extract.

**Figure 3:**
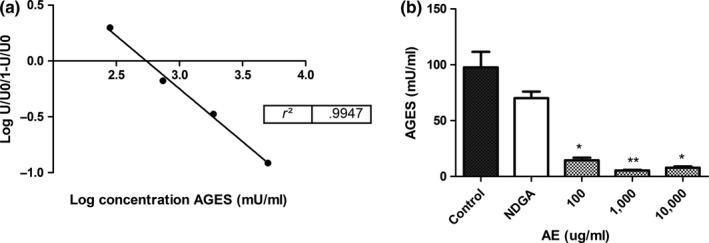
Effect of AE on AGE formation in egg during cooking. The inhibition of AGE formation in eggs during cooking was determined in the absence (control) and in the presence of the extract; this effect was a measure of the antiglycant activity. (a) Calibration curve (b) AGE (mU/ml) in the absence or presence of the extract or NDGA (3 μg/ml). Values were calculated by interpolation in a calibration curve. Results were expressed as mean ± SEM of three determinations made in duplicate. *P < 0.05; **P < 0.01, significant differences with respect to control (ANOVA+ Dunnett's test)

Moreover, the AE was capable of inhibiting AGE formation in egg, cooked by frying, nearly 90% (Figure 3a). As it can be seen in Figure 3b, NDGA, assayed at 3 μg/ml, slightly decreased AGE formation. The antiglycation activity also could be related to polyphenols but not with NDGA. NDGA, at 3 μg/ml (concentration present in 1,000 μg/ml of extract), slightly decreased AGE; therefore, other compounds could be related to this action. It has been demonstrated that the family of phenolic compounds possess significant glycation inhibitory activity both in vivo and in vitro. Not only can polyphenols inhibit the proceeding of the advanced oxidative glycation stage via free radical scavenging capacity (Lunceford & Gugliucci, 2005) but also they can directly trap reactive carbonyl species through adducts formation (Lo et al., 2006; Shao et al., 2008).

It is important to note that the inhibition of MDA formation induced by the extract could explain the anti‐AGE action on eggs during cooking. It is known that MDA is implicated in AGE formation as it is highly reactive and that its reactivity is mainly based on its electrophilicity making it strongly reactive toward nucleophiles, such as basic amino acid residues (i.e., lysine, histidine, or arginine). So, initial reactions between MDA and free amino acids or protein generate Schiff‐base adducts and consequences AGE (Pizzimenti et al., 2013; Traverso et al., 2004).

**Figure 4:**
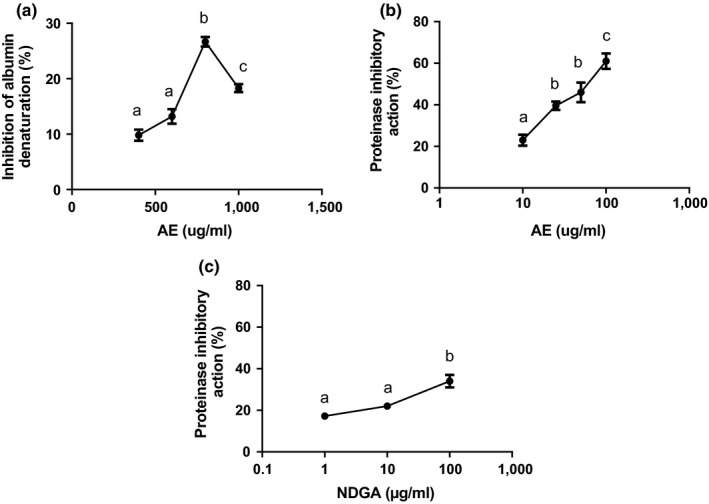
Effect of AE and NDGA on albumin denaturation and on casein trypsin‐induced hydrolysis. Results expressed as inhibition of albumin denaturation (%) or as proteinase inhibitory action (%) represent mean ± SEM of three determinations made in duplicate. a, b, c, and d significant differences among values in accordance with ANOVA plus Tukey test

Moreover, the capacity of the extract to inhibit protein hydrolysis by heat and protease was studied. It can be seen in Figure 4a that the extract could prevent albumin hydrolysis from heat and salicylic acid induces hydrolysis at high concentrations in a concentration–response fashion reaching a maximum value of inhibition at 800 μg/ml. Also, the extract could prevent casein hydrolysis induced by trypsin, in a concentration–response manner (Figure 4b). NDGA did not exert protection to albumin denaturation at 1, 10, and 100 μg/ml (data not shown) but exerted inhibition to casein trypsin‐induced hydrolysis in a concentration‐response manner (Figure 4c).

Proteins are macromolecules constituted by amino acids which possess many nutritional properties. For example, milk is a food that possesses many proteins in its composition such as casein and albumin. But these proteins can be modified (coagulated or precipitated) by actions and agents such as change pH, salts, heat, and proteolytic enzymes (Walstra, Wouters, & Geuters, 2006). The main affected protein because their complexities are caseins. As well, enzymes participate in production and maturation of cheese and yogurt are responsible of many undesirable effects such as off flavors and physic and chemical damage such as sedimentation and gelification during storage (Collins, Buster, & Mc Gill, 1993). It is known that many proteases, which are produced by microorganism and attempt against food preservation, are thermo‐resistant, so other methods are necessary to inhibit them.

It is important to note that the extract possesses beneficial effects for health as it exerts antitumor activity on lymphoma cell lines; in this action, flavonoids are involved (Martino et al., 2013, 2016).

What is more, the extract does not possess acute or subchronic toxicity (Peralta et al., 2015). So these facts become it into a good candidate to be used as a food preserver.

## 4 CONCLUSIONS

These results suggested the potential value of *L. divaricata* extract as a safe source of bioactive compounds such as polyphenols with antioxidant and antiglycation activities. These properties render the extract a good supplement to be incorporated in human and animal food with preservative actions.

## CONFLICT OF INTERESTS

There are no conflict of interests.
